# Effects of a computerized feedback intervention on safety performance by junior doctors: results from a randomized mixed method study

**DOI:** 10.1186/1472-6947-13-63

**Published:** 2013-06-04

**Authors:** Sabi Redwood, Nothando B Ngwenya, James Hodson, Robin E Ferner, Jamie J Coleman

**Affiliations:** 1 College of Medical and Dental Sciences, University of Birmingham, Birmingham B15 2TT, UK; 2 University Hospitals Birmingham NHS Foundation Trust, Edgbaston, Birmingham B15 2SW, UK

**Keywords:** Patient safety, Clinical decision support, Junior doctors

## Abstract

**Background:**

The behaviour of doctors and their responses to warnings can inform the effective design of Clinical Decision Support Systems. We used data from a University hospital electronic prescribing and laboratory reporting system with hierarchical warnings and alerts to explore junior doctors’ behaviour. The objective of this trial was to establish whether a Junior Doctor Dashboard providing feedback on prescription warning information and laboratory alerting acceptance rates was effective in changing junior doctors’ behaviour.

**Methods:**

A mixed methods approach was employed which included a parallel group randomised controlled trial, and individual and focus group interviews. Junior doctors below the specialty trainee level 3 grade were recruited and randomised to two groups. Every doctor (N = 42) in the intervention group was e-mailed a link to a personal dashboard every week for 4 months. Nineteen participated in interviews. The 44 control doctors did not receive any automated feedback. The outcome measures were the difference in responses to prescribing warnings (of two severities) and laboratory alerting (of two severities) between the months before and the months during the intervention, analysed as the difference in performance between the intervention and the control groups.

**Results:**

No significant differences were observed in the rates of generating prescription warnings, or in the acceptance of laboratory alarms. However, responses to laboratory alerts differed between the pre-intervention and intervention periods. For the doctors of Foundation Year 1 grade, this improvement was significantly (p = 0.002) greater in the group with access to the dashboard (53.6% ignored pre-intervention compared to 29.2% post intervention) than in the control group (47.9% ignored pre-intervention compared to 47.0% post intervention). Qualitative interview data indicated that while junior doctors were positive about the electronic prescribing functions, they were discriminating in the way they responded to other alerts and warnings given that from their perspective these were not always immediately clinically relevant or within the scope of their responsibility.

**Conclusions:**

We have only been able to provide weak evidence that a clinical dashboard providing individualized feedback data has the potential to improve safety behaviour and only in one of several domains. The construction of metrics used in clinical dashboards must take account of actual work processes.

**Trial registration:**

ISRCTN: ISRCTN72253051

## Background

Computer-based systems are widely advocated as one of the most effective means of improving patient safety [1,2]. As the prescribing of medications is a large contributor to risk to patient safety there is much scope for improvements by reducing error in the prescribing process [3]. Electronic prescribing has been shown to reduce medication errors and adverse drug events [4], and improve practitioner safety performance [5]. Monitoring the use of a Clinical Decision Support System (CDSS) is an important strategy in its successful implementation and adoption [6]. We sought to use the capability of such a system to monitor individual doctors’ prescribing activity and their responses to alerts (low level pop-up messages providing important information about an individual patient within their record), and to the two types of warnings (on-screen messages during order entry, giving safety information, and high level pop-up messages providing important information about an individual patient within the system). As junior doctors perform the majority of prescribing actions in UK hospitals [7], we focused on their practice. Junior doctors in this study are defined as doctors who are below the specialty trainee level 3 grade.

Performance dashboards are computer-generated visual representations of numerical data in the form of calibrated dials intended to provide the feedback needed to effect change within an organisation [8]. Dashboards can be divided into 3 distinct types: 1) operational – used to monitor processes, 2) tactical – for monitoring and analysing processes, and 3) strategic – for monitoring strategic objectives [8]. Dashboards have emerged as a helpful tool for hospital directors to improve the quality of care. However, most display clinical quality measures rather than patient safety measures and provide high level data to executives rather than individual level specific data for performance management [9]. Although clinical dashboards are now widely implemented across the National Health Service (NHS) [10], there is no evidence on the use of dashboards for junior doctors at a day-to-day operational level.

Performance measurement and feedback are crucial elements in identifying problems and helping individuals improve their performance [11,12]. Providing feedback to junior doctors offers insight into their clinical practice and prescribing behaviours, giving them the opportunity to learn from potential ‘near misses’ that are indicated by warnings generated by a CDSS, and make them aware of how they compare with the safe level of competence. This can be the catalyst needed to modify individual behaviour [13].

We undertook a randomized controlled trial of a dashboard for operational use at the individual level which compares doctors to their peers. Individual feedback on actions performed within the system was presented to the intervention group while the control group received no such feedback. The purpose of the study was to determine whether the widespread use of operational clinical dashboards could be implemented at individual level with equal success. Our hypothesis was that junior doctors would improve their response to computer prompts when feedback indicates that they are performing poorly by generating more prescription warnings and/or ignoring more alarms/alerts, compared to their peers.

## Methods

### Setting and study population

The study was carried out in a large NHS Foundation Trust teaching hospital. The hospital has a locally-developed electronic prescribing system known as PICS (Prescribing, Information and Communication System), which is used across all (approximately 1200) inpatient beds. The system covers all general and specialist medical and surgical wards. There are no inpatient beds for obstetrics, paediatrics, or mental health. PICS has a comprehensive audit database of administrative, prescribing and drug administration transactions within the system, including messages displayed to users and actions taken in response. Within PICS, the prescription warnings generated are hierarchically classified according to a decreasing level of seriousness: (1) disallow warning (this is a ‘hard stop’ warning with the associated on-screen text ‘prohibited action – unable to proceed’); (2) password warning (this is an interruptive warning with on-screen advice requiring the user to provide their password in order to proceed with the action); (3) tick box warning (this in a non-interruptive warning requiring acknowledgement by clicking onto a tick box); and (4) warning to consider additional safety information (this warning provides information with the associated on-screen text ‘no further action required’).

The system also alerts prescribers to the presence of abnormal laboratory values. When abnormal laboratory values are received into the system, pop-up alerts are generated which are visible to junior doctors logged into the system in the relevant specialty. In case of seriously abnormal laboratory results, interruptive alarms are generated. When responding to alerts or alarms, doctors can either click a button on the electronic system to “accept” the message (and thus show explicitly that they have acknowledged the clinical implications of the decision to proceed), or click a button to “ignore” the message. Junior doctors are explicitly told that unless there are good reasons all warnings should be considered carefully and acknowledged where action needs to be taken. We are using acceptance rates as a surrogate for responsible action. However, it is possible that junior doctors would ignore warnings on the system, yet still take appropriate clinical action. This cannot be controlled for within this type of research. While it is possible that junior doctors will ‘accept’ a warning to move past it, rather than click the ‘ignore’ button, this is less likely to happen because all individual actions are logged on the system and stored, and thus capable of being traced back to an individual. Accepting an alert without considering the clinical implication constitutes a violation against good medical practice which carries serious sanctions which in turn is likely to deter doctors from doing so.

Junior doctors below the specialty trainee level 3 (within about four years of graduation and equivalent to junior residents) who had used PICS for four months preceding the trial, and who would be using the system during the trial period were eligible for inclusion. We excluded doctors who had issued fewer than 10 prescriptions in the previous four months, and those who had only recently joined the hospital and for whom no baseline data were available. Given that we were randomising a specific cohort of junior doctors, the maximum sample size and trial duration were fixed. For this reason, a sample size calculation was not carried out initially. However, power calculations were performed on the data collected prior to the intervention.

According to the hospital records, 229 junior doctors were within the eligibility criteria and received an information sheet and consent form through the hospital e-mail system, asking them to participate in the trial. A reminder e-mail was sent four weeks later. In addition, we attended junior doctors’ teaching sessions to publicise the trial, and displayed posters and information leaflets in clinical areas. One hundred and thirty nine doctors were excluded as it later transpired that their employment records had not been updated and they were above level 3 training. Data from junior doctors in their first year after graduation Foundation Year 1 (FY1) were analysed separately, because we hypothesized a priori that due to their limited clinical experience they would behave differently to those doctors with greater clinical experience and therefore show greater effects.

### Ethics and research governance

A favourable ethical opinion was given by the NHS East Midlands Nottingham 1 research ethics committee. Permissions were obtained by the local Trust Research and Development department. Signed consent was obtained from all participants upon entry into the trial. Additional signed consent was taken prior to individual or focus group interviews.

### Randomisation

An independent statistician randomly assigned the doctors in the trial to the intervention and control groups using the random number function in Microsoft Excel. In order to ensure that the different grades of doctor were equally represented in the control and intervention groups, the group assignment was stratified by the doctor grade. This resulted in the following stratification:

Twenty three FY1 doctors and 21 other JDs in the intervention group, whilst the control group had 22 FY1 doctors and 22 other JDs.

### Intervention

**Figure 1:**
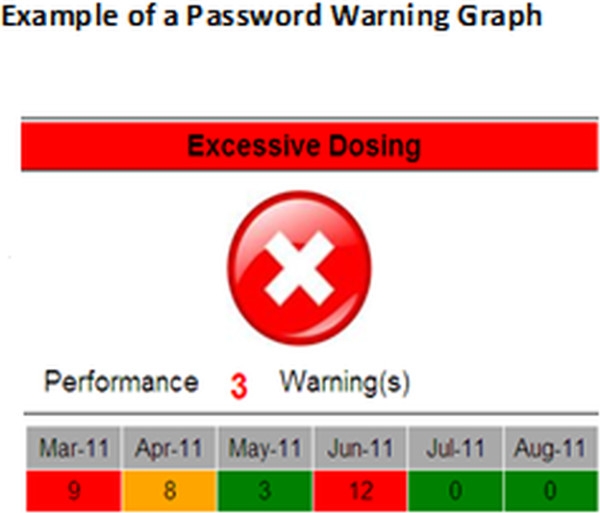
**Example of a password warning graph.** The prescription warning information displays 6 dials for disallowed and password warnings for each category of allergy/contraindications (combined), excessive dosing, and drug interactions.

**Figure 2:**
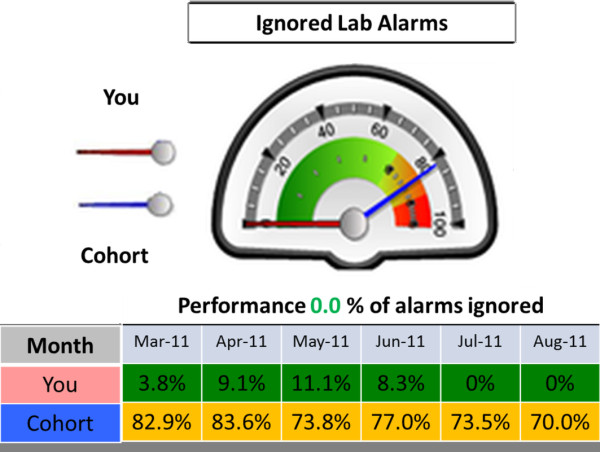
**Example of a laboratory alarm dial.** The laboratory alerting section contains 2 dials with the percentage of ignored alarms and alerts.

We used the PICS database to develop the Junior Doctors’ Dashboard (JDD), based on the two highest warning levels for prescribers– disallow and password warnings – which indicate that there is potential for patient harm. The two lower level warnings are frequently associated with more trivial information, rarely relate to patient harm and were not considered further. Information from the PICS database was pre-aggregated for the junior doctors in the trial for the period under investigation and presented in the form of dials, charts and graphs for each doctor. JDD has 8 dials: 6 dials show prescription warning information (Figure 1) and 2 dials show laboratory alerting acceptance rates (Figure 2). For 4 months from the beginning of April 2011, we sent each participant in the intervention group weekly e-mails with a link to his or her unique individual dashboard.

### Quantitative data collection and analysis

Outcomes for prescription order warnings (password or disallow level warnings) and laboratory alerts and alarms (message ignored and signed off) were collected for the four months immediately preceding the intervention, and for the subsequent four months. Each prescription ordered, and each laboratory alert or alarm displayed during this period was attributed to a junior doctor and then numbered chronologically. For each record, it was then determined whether an outcome under consideration had occurred. There were four such outcomes, namely generating either password or disallow warnings during a prescription, and ignoring a laboratory alarm or alert. Each of these outcomes was analysed separately for junior doctors of FY1 grade and of more senior grades, based on the assumption that the level of clinical experience would affect the behaviour of junior doctors. This resulted in a total of eight analyses.

A power calculation which took into account within-doctor correlation found the detectable difference in the rate of ignoring password warnings to be < 10% for both grades of doctor (at 80% power with 5% alpha). All of the other outcomes considered were adequately powered to detect rate reductions of around a third, with the exception of alerts and alarms for the more senior grade of doctor, which had larger detectable differences due to the observed rates being considerably lower than in FY1s.

During the power calculation, non-trivial levels of correlation were detected in the doctors’ responses to laboratory alerts and alarms. In order to account for this, the analyses were performed using generalised estimating equations with an exchangeable correlation structure [14]. This controlled for the potential non-independence of repeated measures on the same junior doctor. Binary logistic models were used, with the dependent variable being whether a warning was generated at the relevant level for the prescribing data, and whether a message was ignored for laboratory alert and alarm data.

Each of the analyses included the same set of three predictor variables. The first two factors specified whether each prescription, laboratory alert or laboratory alarm occurred before or after the intervention commenced, and whether the junior doctor generating the prescription or being shown a laboratory alert or alarm was in the control or intervention group. An interaction term between these two factors was also included. This was the key variable in the model, as it tested whether any change that occurred after the intervention differed significantly between the group who had access to the dashboard, and the control group.

Since several outcomes were being tested for each doctor grade, the critical p-value was corrected for multiple comparisons. A total of eight analyses were carried out, hence the the critical p-value of 0.05 was Bonferroni-adjusted to 0.00625. All analyses were performed using SPSS v19.0.0 (IBM SPSS Inc., Chicago).

### Qualitative data collection and analysis

In June and July 2011, we invited participants from the intervention and control groups to participate in either an individual or focus group interview, depending on their availability. This qualitative phase of the study was conducted after the trial to help give a better understanding of the quantitative results. All participants were contacted through their work e-mail. Interviews and focus groups lasted 1 to 1.5 hours depending on the number of attendees. The aim was to explore participants’ experiences, opinions, beliefs and concerns regarding the use of the dashboard (if they were in the intervention group), and regarding their use of PICS. The semi-structured individual interviews [15] were organised around a set of open-ended questions about their experience with the dashboard and/or PICS. The aim of group interviews was to facilitate interactions between participants as a way of collecting rich data. Participants respond not only to the researcher, but also to each other, producing new ideas, questions and priorities. As such, both critical comments and solutions to problems were likely to be generated during discussions [16]. The focus groups were facilitated by researchers who were neither directly involved in supervising the doctors or active users of the PICS system. A discussion guide was used to ensure that key topics were covered during the group interview. Both individual and focus group interviews were audio-recorded with participants’ consent and transcribed.

Interview transcripts were checked for accuracy against the original recordings. Data were coded with the aid of the QRS Nvivo^®^8 qualitative data management software, and analysed inductively. The constant comparative method developed by Glaser and Strauss [17] was used with systematic efforts to check and refine emerging categories of data. Open codes were generated by two researchers (NN and SR) and developed into higher order thematic categories, to develop a flexible analytic framework. The framework was modified as new data were collected and coded in order to delineate emerging categories and identify relationships between them. Given the specificity of the evaluation, the homogeneity of the participants, and the quality of the individual interview and focus group data, saturation (the point at which collecting additional data neither adds new information nor requires revisions to be made to findings already developed) was reached.

## Results

**Figure 3:**
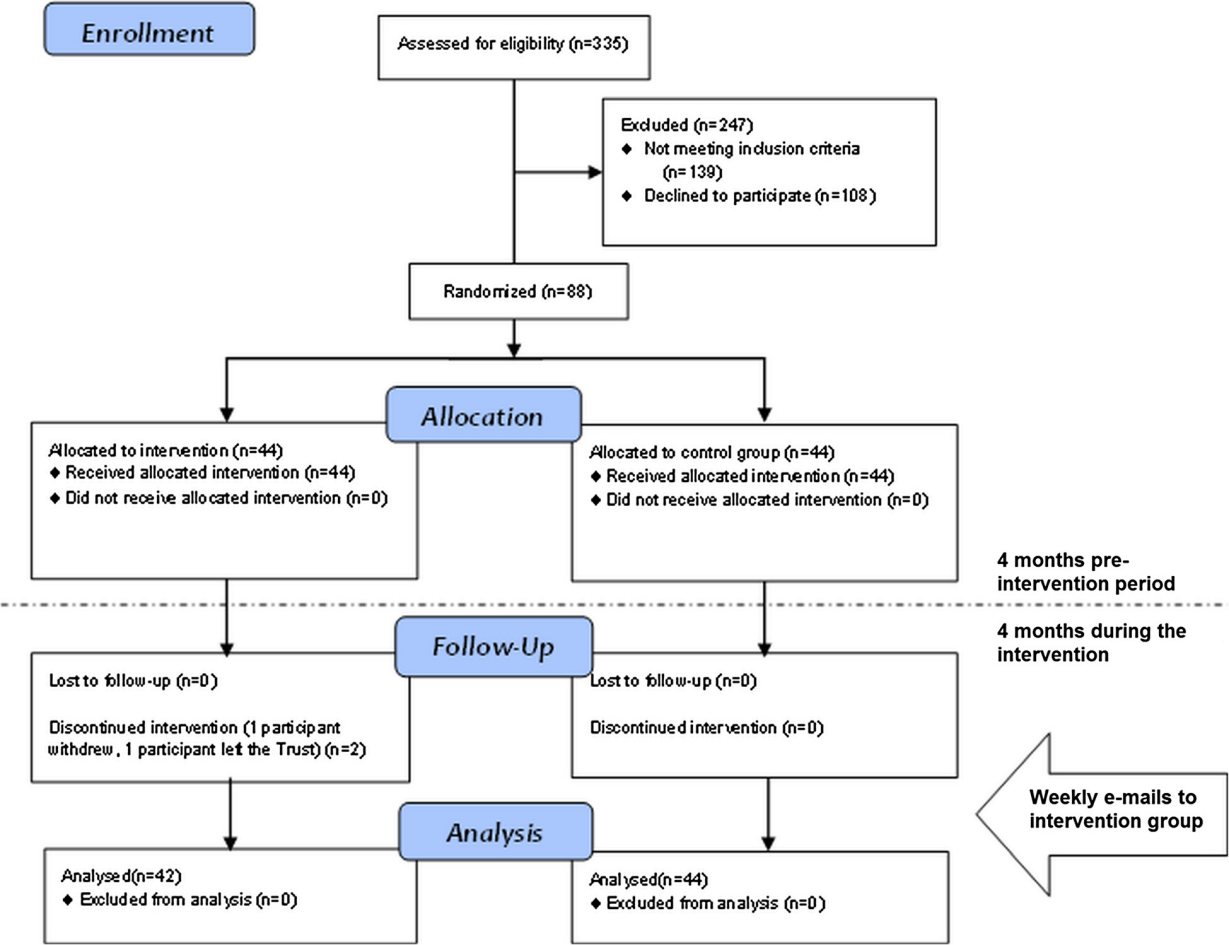
**Flow of participants through trial.** This figure adheres to the consort statement and shows the phases of recruitment for the trial.

Figure 3 shows the flow of participants throughout the RCT. The sample consisted of 88 participants (45 men and 43 women), 44 in each of the two parallel groups (intervention and control). The results of the generalised estimating equations are summarised in Table 1.

**Table 1:** Table of results

**Outcome**	**Doctor grade**	**Significance of**		
		**Group**	**Pre/Post intervention**	**Interaction**
Laboratory Alert	FY1	0.498	<0.001*	0.002*
Laboratory Alert	Other JD	0.386	0.005*	0.977
Laboratory Alarm	FY1	0.004*	0.024^§^	0.587
Laboratory Alarm	Other JD	1.000	0.173	0.341
Password Warning	FY1	0.981	0.371	0.774
Password Warning	Other JD	0.991	0.940	0.671
Disallow Warning	FY1	0.932	0.420	0.890
Disallow Warning	Other JD	0.894	0.641	0.643

*Significant at p < 0.05 and after Bonferroni Correction for eight comparisons (p < 0.00625).

^§^Significant at p < 0.05, but not after Bonferroni Correction for eight comparisons (p < 0.00625).

Table 1 reports the p-values of the factors in the generalised estimating equations. No factors were found to be significant for the prescribing outcomes. Similarly, for the two outcomes relating to laboratory alarms, neither the change from pre- to post-intervention, nor the interaction terms were found to be significant (after correction for multiple comparisons). Hence, it can be concluded that there is no evidence that the introduction of the dashboard had a significant effect on either the prescribing behaviour, or the response to laboratory alarms of the junior doctors in the trial.

Conversely, the response to laboratory alerts was shown to change significantly after the introduction of the dashboard. For doctors of FY1 grade, the proportion of ignored alerts fell from 51% to 39% (p < 0.001) after the intervention, with a change from 38% to 31% (p = 0.005) observed in junior doctors of other grades.

**Figure 4:**
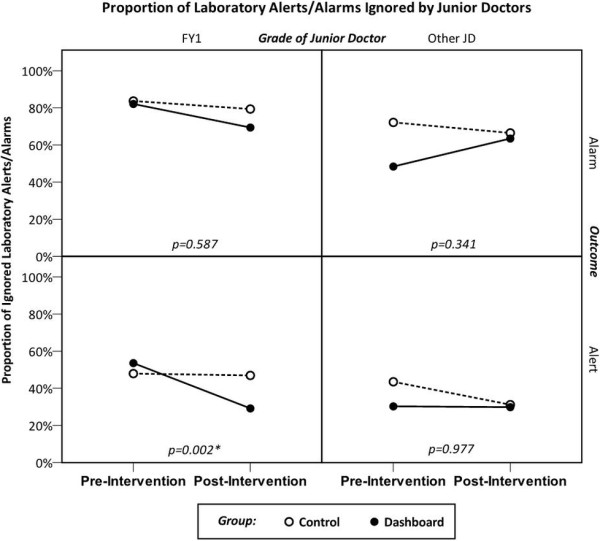
**Results of alerts and alarms ignored by junior doctors.** Plot of the proportion of laboratory alerts and alarms ignored by the junior doctors during the trial. The quoted p-values are those relating to the interaction terms in the generalised estimating equations.

The interaction term in the model tests whether these improvements were significantly larger in the group with access to the dashboard than in the control group. The analysis of the more senior group of doctors did not have a significant interaction (p = 0.977), giving no evidence that the junior doctors with access to the dashboard showed a greater improvement in the rate of ignored laboratory alerts than those in the control group. However, the analysis of the junior doctors of FY1 grade did yield a significant interaction, with p = 0.002. Figure 4 illustrates this finding. For doctors in the control group, the intervention coincided with a small reduction in the rates of ignored laboratory alerts from 47.9% to 47.0%. Over the same period, the improvement in the group with access to the dashboard was considerably larger, with a decrease from 53.6% to 29.2%.

### Qualitative findings

A total of 19 doctors participated in two focus groups and nine one-to-one interviews. Eleven interviewees were drawn from the intervention group, and eight from the control group. We organised the data into four broad themes on (1) perceptions on clinical decision support, (2) feedback on performance, (3) limits of clinical decision-making and (4) appropriate accountability. Interview excerpts for each theme are provided in Table 2. Perceptions on clinical decision support encompassed a wide range of views on the clinical useful and relevance of PICS to the work of junior doctors. While the electronic prescribing functions were positively evaluated in enhancing patient safety and reducing the amount of time spent on checking safety information, junior doctors were discriminating in the way they responded to other alerts and warnings given that from their perspective these were not always immediately clinically relevant or within the scope of their responsibility. With regard to feedback on their performance, junior doctors found the dashboard helpful in stimulating reflection on their clinical behaviours and responsibilities. However, they expressed reservations about the sort of performance data that were collected and given as feedback via the JDD. Junior doctors are learners in the clinical environment and the data indicate that the constraints placed on their clinical independence means that they often carry out actions that have been ordered by senior doctors. Thus junior doctors perceived the feedback provided via the dashboard to be an inaccurate account of their own clinical practice given that actions that generate alarms, alerts and warnings in PICS against their log-on IDs had not necessarily been initiated by them, but ordered by senior doctors. Furthermore, doctors’ priorities change in emergencies, and they are less likely to sign off alerts. In other instances there are limitations due to systemic constraints which do not allow for flexibility in prescribing practices in specific clinical contexts where deviation from recommended doses may be clinically indicated.

**Table 2:** Table of interview excerpts

**Theme**	**Interview excerpts**
Perceptions of clinical decision support	*“The alerts and warnings although frustrating at times are very useful in the flagging of things that you may not have seen or thought of and yeah, just the in-built prescribing for certain drugs for particular dosages, which it suggests, obviously that’s great it makes our life a lot easier, we’re not always looking in the BNF (British National Formulary).”* (Individual Interview 9)
	*“The way I use PICS (Patient Information and Communication System) is quite safe and okay there were some alerts which or the warnings that I ignored but there, I wouldn’t ignore say a red SEWS (Standardised Early Warning Score) score box but I would ignore the thrombosis assessment because the patient is on Enoxaparin.”* (Individual interview 3)
	*“I find that it’s quite overwhelming to log on to the system and suddenly see alert after alert (…) but I’ve had a couple of occasions where I’ve been on call and I’ve had a flash-up of an alert on a patient’s observation and it hasn’t been communicated to me in any way by any of the nursing staff and I’ve been able to go and see that patient and find that actually PICS is alerting me to this patient and actually they are quite unwell and probably should have been seen so once or twice I’ve found it very useful.” (*Individual Interview 4)
	*“Sometimes you can ignore and sometimes you can accept because sometimes I can’t remember what I was supposed to click (…) because you’re reflecting on what you have to do for the patient.”* (Focus group 2)
	*“Lab alerts and alarms I don’t think are of any relevance whatsoever in my prescribing practice, it doesn’t change the way that I work.”* (Focus group 2)
Feedback on performance	*“I think that’s the only thing that to be honest I kind of took away which was that I was more conscientious about my prescribing. When I see the levels go sort of high that’s when I sort of started making sure my dosing was correct and that the drug history was correct and that I was looking at interactions and was cautious about allergy status so I was just saying as a whole it flags up to me that you know to be actually a bit more cautious.”* (Individual interview 4)
	*“We crave feedback, to know whether we are good doctors or bad doctors or what we’ve done well or what we’ve done badly, but unfortunately the feedback that we desire and the feedback that we get are very different.”* (Focus Group 2)
	*“Military patients have a set pain protocol which involves (…) prescribing a number of opioids. So every time that I put somebody on this pain protocol, I get a red alert saying ‘multiple opioid drugs prescribed, are sure you want to proceed?’, so I tick yes but obviously then on the dashboard I will get a negative mark if you like.”* (Individual Interview 6)
Limits of clinical decision-making	*“Sometimes you do override warnings on PICS for different reasons and that is usually not because you’re being blasé about it but it can sometimes be because you’re on ward round and the boss says ‘prescribe this’ and you’ll say ‘do you know about this’ and he says ‘yes continue’. (…) So it’s not your decision.”* (Individual Interview 8)
	*“A lot of the prescribing decisions are [made by the] consultant…like it’s very unusual that a consultant on a ward round will log into his PICS and prescribe the drug.” (*Individual Interview 5)
	*“Decisions to put patients on drugs isn’t really down to us anyway. I wouldn’t say ‘start a patient on laxatives or painkillers’, but then other than emergency treatment I never really start a patient on drugs by my own means. I will always go through a senior doctor.....So are you looking at the right cohort as to who makes the decisions?”* (Individual Interview 3)
Appropriate Accountability	*“I think when it comes to alerts there should be accountability i.e. if it’s your patient that you’re looking after it’s useful to have an alert ….. if I see someone that I don’t know the patient then I will press ignore. So there should be accountability, but if someone came to me and said ‘I don’t like how many things you’ve been ignoring’ I would say to them ‘I don’t care it’s really not my concern’.”* (Focus Group 1)
	*“Sometimes I think it pressured me into ticking off things that maybe I shouldn’t have been ticking off, particularly when you’re doing say night cover so (…) every ward you get on to you get flashed up a selection of lab alarms about patients you’ve never met so it’s not really appropriate to be accepting those because you don’t know anything about any of them (…) you don’t really feel like that’s my responsibility and yet at the same time, I’m ignoring lab alerts.”* (Individual Interview 2)
	*“Overnight when I do nights and things flash up and it’s in the relevant directorate (…) then clearly I can’t click ‘ignore’ because that is my responsibility so I go and deal with it, whatever that alert might be. But during the day, you know if things start flashing up and it’s not my patient…you know there’s a lot of patients in this hospital. I’m not going to respond to everything…”*(Individual Interview 5)

Some participants expressed concerns about the use of data for surveillance and performance management purposes. They acknowledged the potential benefits that could be derived from capturing responses to warning and alerts and feeding these back to junior doctors to encourage reflection and behaviour modification. However, the fact that such data could be used for audit purposes also produced anxieties about clinical accountability and disciplinary action.

## Discussion

Feedback to junior doctors about aspects of their clinical activity is common practice and prescribing behaviour has received particular attention in various specialties and situations [18-20]. We believe that this is the first trial to examine the value of a dashboard that provides formative clinical performance feedback to junior doctors with the aim of improving their safety performance.

A surprisingly high proportion of laboratory alerts was ignored in the baseline period. This could have reflected concern about taking responsibility for acceptance, but might also have indicated a disregard for safety. The intervention was intended to promote responsibility and safe practice through the use of electronic feedback. The proportion of laboratory alerts ignored by FY1 doctors fell significantly during the intervention, and almost all the change occurred in the intervention group. There was also a significant decrease in the proportion ignored by other doctors, but equally in the intervention and non-intervention groups. This finding is likely to be an example of the Hawthorne effect, a well documented phenomenon in studies evaluating ICT interventions in healthcare settings [21]. However, we also suggest that junior doctors felt that they have sufficient authority to accept the less serious alerts, but left the more serious laboratory abnormalities (alarms) to more senior doctors, including doctors not involved in this study.

Our findings are in line with similar studies which have sought to improve clinicians’ practice through the use of computer mediated feedback and demonstrated only small to moderate effect [22]. While changes in our study were significant in only one of four domains, the results suggest that providing information to junior doctors about electronically captured data on their practice has the potential to change their behaviour. This assumes that the reduction in ignoring such alerts by accepting responsibility for following up on abnormalities in laboratory results is contingent on the feedback they received. Although we acknowledge that modest levels of behaviour change will not automatically translate into actual improved practitioner performance and the delivery of safer and higher quality care [3], further development of the tool and refinement of the feedback metrics offer the possibility of a more detailed evaluation of its impact on patient safety.

The role of CDSS in improving practitioner performance with regard to the ordering and review of laboratory tests is well established [23]. It has also been suggested that patient outcomes and safety can be improved through electronic endorsement to indicate the appropriate follow-up of test results in the electronic delivery of laboratory results [24]. Furthermore, studies investigating timely clinical responses to abnormal laboratory result warnings highlight the value of creating warnings that provide temporal information. The authors argue that by showing what is missing and how much time has passed since the last action, responsibility can be attributed more easily [25]. Introducing such functionalities into CDSS and subsequent feedback dashboards, we suggest, may enhance junior doctor safety performance.

While junior doctors were positive in principle about receiving feedback about their clinical performance and safety behaviours, the measured effectiveness of JDD was low. This was partly because recipients judged the metrics used to feed back performance data not to be a fair reflection of their own clinical competence. Qualitative findings from interviews with junior doctors underlined the fact that in order for a dashboard to be effective, the metrics used need to be concrete rather than abstract and must reflect actual work processes which may be different in different clinical contexts (e.g. working on a day or night shift, or in a surgical or medical speciality). Feedback on metrics over which junior doctors have no behavioural control is likely to lead to disengagement with the process. If junior doctors feel that they cannot be held directly accountable for the feedback that is generated for them, the feedback will lack the ‘moral authority’ to lead to changes in behaviour. Interventions to improve feedback for JDDs will require further development to ensure they are sufficiently tailored to the recipients’ level of clinical responsibility and specifically adapted to the clinical context in which they work. Central to such improvements will be to refine alerts and warnings to promote compliance and avoid alert fatigue [26]. Further research is needed to clarify how information should be presented, in terms of information provided, place on the screen, format and colour [27,28]. Personalised feedback has also been shown to improve patient safety [29] while the timing, format and the value of the information provided are crucial to eliciting compliance [12,30]. In relation to junior doctors in particular, CDSSs may need to be engineered to take account of their lack of biomedical and experiential knowledge base and their limited expertise in framing clinical situations [31]. The effectiveness of feedback in other circumstances depends upon baseline performance, repetition, clear targets, and other factors [32]. It may be that doctors will respond more effectively to computer feedback presented in other ways and with explicit suggestions for improvement, as could be included in an automated dashboard commentary. It may also be that more personal feedback, for example, by email from the supervising senior doctor, would be more effective.

The findings also suggest concerns about the ubiquitous panoptic gaze on clinical practice. Junior doctors expressed some anxiety about the surveillance and auditing of their clinical practice and a lack of certainty about what use this ‘dataveillance’ [33], was being put to. While they supported the electronic capture of their data to stimulate reflective practice and clinical learning, [34] they were anxious that such data could be employed to performance manage and discipline them.

### Strengths and limitations of study

The strengths of this study are the triangulation of methods and randomized trial design. Efforts were made to minimise bias including the randomisation, study participants and analyses although we acknowledge that there were observed differences between intervention and control groups at baseline despite the randomisation process. However, the findings need to be interpreted in the context of certain limitations. First, the sample may have been biased towards volunteers who were already motivated and safety conscious. Second, information on the junior doctors’ demographics was not taken into account in the analysis which may therefore have contained hidden confounding factors. Third, it was not possible to conduct a blinded randomisation due to the nature of the intervention. Fourth, the trial was dependent on junior doctors accessing the JDD to initiate change in behaviour. However, we did not set out to monitor individual participants’ adherence to accessing the dashboard. Finally, caution must be exercised in any attempt to generalise the findings, given the sample size and the specific attributes of the PICS system. The decision support rules that work in the system relating to critical laboratory results and prescription warnings are locally developed with the clinical teams, and whilst care is taken to ensure that these are sensitive and specific to potential harms, the system set-up and user interface may not be optimal for testing junior doctor behaviour.

## Conclusion

Computerized health care environments— and CDSSs in particular— make it possible to monitor individuals’ responses to warnings generated by their own prescribing activities, and to alerts activated by abnormal laboratory results. We have only been able to provide weak evidence that a clinical dashboard providing individualized feedback data has the potential to improve safety behaviour and only in one of several domains. However, a number of issues have to be taken into consideration when transforming routine monitoring data into an effective educational intervention to improve junior doctors’ safety behaviour. The construction of metrics used in clinical dashboards needs to take account of actual work processes which may be different in different clinical contexts. Feedback on performance must also be credible and reflect actual clinical behaviour to ensure individuals are accountable for the data they generate in the system. Furthermore, it must be within the gift of those receiving feedback to change their behaviour. With appropriate technology, design and metrics in place the JDD can be incorporated into routine practice and assist doctors in developing safe clinical and prescribing practices.

## Competing interests

The authors declare that they have no competing interests.

## Authors’ contributions

JJC was the principal investigator and developed the idea for the study. JJC, NN and JH designed and coordinated the study design. NN carried out data collection. JH carried out the statistical analyses. JJC, NN & SR analysed the qualitative data and prepared the first draft of the manuscript. REF chairs the management committee overseeing the research group and provided helpful advice during the study. NN prepared the initial manuscript and all authors commented on multiple drafts. SR prepared the final version of the manuscript which was agreed by all authors.

## Pre-publication history

The pre-publication history for this paper can be accessed here:

http://www.biomedcentral.com/1472-6947/13/63/prepub
